# An explorative analysis on the optimal cryo-passes and freezing time of the ultrathin cryoprobe in endobronchial ultrasound-guided transbronchial mediastinal cryobiopsy

**DOI:** 10.1038/s41598-024-69702-y

**Published:** 2024-08-12

**Authors:** Sze Shyang Kho, Shirin Hui Tan, Chun Ian Soo, Hema Yamini Devi Ramarmuty, Chan Sin Chai, Nai Chien Huan, Khai Lip Ng, Yuji Matsumoto, Venerino Poletti, Siew Teck Tie

**Affiliations:** 1 Division of Respiratory Medicine, Department of Medicine, Sarawak General Hospital, Jalan Hospital, Ministry of Health Malaysia, Kuching, Sarawak Malaysia; 2 Clinical Research Centre, Sarawak General Hospital, Institute for Clinical Research, National Institutes of Health, Ministry of Health Malaysia, Kuching, Sarawak Malaysia; 3 Division of Respiratory Medicine, Department of Medicine, University Malaya Medical Centre, University Malaya, Kuala Lumpur, Malaysia; 4 Department of Respiratory Medicine, Queen Elizabeth Hospital, Ministry of Health Malaysia, Kota Kinabalu, Sabah Malaysia; 5 Respiratory Endoscopy Division, Department of Endoscopy, National Cancer Center Hospital, Tokyo, Japan; 6 Department of Medical Specialities-Pulmonology, GB Morgagni Hospital, Forlì, Italy; 7 Department of Medical and Surgical Sciences (DIMEC), Bologna University, Bologna, Italy

**Keywords:** Endobronchial ultrasound, Mediastinal cryobiopsy, Freezing time, Cryo-pass, Transbronchial needle aspiration, Cancer imaging, Lung cancer, Cancer imaging

## Abstract

EBUS-guided transbronchial mediastinal cryobiopsy (TBMC) has emerged as a promising biopsy tool for diagnosing hilar and mediastinal pathologies. However, several fundamental technical aspects of TBMC remain unexplored. This study aims to determine the optimal number of cryo-passes and freezing time of the ultrathin cryoprobe in EBUS-TBMC concerning specimen size and procedural diagnostic yield. We conducted a retrospective chart review of patients with mediastinal and hilar lesions who underwent EBUS-TBMC between January 2021 and April 2023 across three hospitals in Malaysia. A total of 129 EBUS-TBMC procedures were successfully completed, achieving an overall diagnostic yield of 88.4%. Conclusive TBMC procedures were associated with larger specimen sizes (7.0 vs. 5.0 mm, *p* < 0.01). Specimen size demonstrated a positive correlation with diagnostic yield (*p* < 0.01), plateauing at specimen size of 4.1–6.0 mm. A significant positive correlation was also observed between the number of cryo-passes and both specimen size (*p* < 0.01) and diagnostic yield (*p* < 0.05). Diagnostic yield plateaued after 2–3 cryo-passes. In contrast, longer freezing times trended towards smaller specimens and lower diagnostic yield, though not reaching statistical significance. The highest diagnostic yield was recorded at the 3.1–4.0 s freezing time. The safety profile of TBMC remains favourable, with one case (0.8%) of pneumothorax and nine cases (7%) of self-limiting bleeding. In our cohort, TBMC performance with 2–3 cryo-passes and a 3.1–4.0 s freezing time to achieve a total aggregate specimen size of 4.1–6.0 mm appeared optimal. Further prospective studies are needed to validate these findings.

## Introduction

Endobronchial ultrasound guided transbronchial needle aspiration (EBUS-TBNA) is a revolutionary diagnostic tool in the field of interventional pulmonology^1,2^. It allows precise access to mediastinal, hilar, and peribronchial structures—traditionally only reachable via invasive techniques such as mediastinoscopy^1^. These advantages, coupled with an excellent safety profile, solidifies EBUS-TBNA as the recommended tool for diagnosis and staging of lung cancer according to major guidelines^3,4^.

However, beyond its established role in lung cancer staging and in diagnosing common malignancies, the application of EBUS-TBNA in uncommon tumours such as lymphoproliferative disorders or benign entities such as sarcoidosis remains a subject of debate^5^. TBNA primarily provides cytology or cell block samples, which may be insufficient for pathologies with complex morphology which frequently requiring sophisticated immunohistochemical or molecular analyses^6,7^. Different techniques have been described to enhance the diagnostic capabilities of EBUS-TBNA, from using core biopsy needles to exploring different EBUS needle types and sizes as well as performing intra-nodal forceps (IFB) biopsies^8–10^. However, small sample sizes and crushed artefacts are among the main drawbacks of these supplementary techniques.

The idea of acquiring larger mediastinal samples with preserved tissue architecture has catapulted the idea of performing EBUS-guided transbronchial mediastinal cryobiopsy (EBUS-TBMC). EBUS-TBMC was made possible with concurrent miniaturization of flexible cryoprobes, allowing insertion of the 1.1 mm ultrathin cryoprobe via the EBUS working channel directly into mediastinal targets under real time ultrasound vision. The first description of EBUS-TBMC was back in 2020, followed by rapid emergence of multiple case reports and case series from various regions worldwide^11–15^. Subsequent randomized controlled trials have demonstrated the feasibility and safety of EBUS-TBMC, highlighting its superiority, particularly in the diagnosis of uncommon tumours and benign disorders^16–18^.

Nevertheless, a few fundamental technical aspects of EBUS-TBMC are still unanswered. To address these knowledge gaps, we performed an explorative analysis on the optimal number of cryo-passes and freezing time, and their impacts on the specimen sizes and diagnostic yield in EBUS-TBMC procedures. As a secondary endpoint, we also aimed to assess the efficacy and safety of EBUS-TBMC in a *real-worl*d setting.

## Methods

### Study design and setting

This retrospective, multi-centre study was conducted across three hospitals in Malaysia, including 2 in the island of Borneo: Sarawak General Hospital (*SGH*) in the region of Sarawak and Queen Elizabeth Hospital (*QEH*) in Sabah. The third hospital, University Malaya Medical Centre (*UMMC*), is in the state of Selangor, Peninsular Malaysia. The study spanned a total duration of 28 months, from January 1, 2021, to April 30, 2023. Adhering to the principles of the Declaration of Helsinki, the study obtained approval from the Medical Research and Ethics Committee, Ministry of Health Malaysia [*NMRR-ID-23–00,577-6XP (IIR)*, *dated June 2, 2023*] in which individual consent for this study was waived according to the approved protocol.

### EBUS guided transbronchial mediastinal cryobiopsy (EBUS/TBMC)

#### Pre-procedural planning and case selection

Patients selected for EBUS-TBNA were evaluated for any potential contraindications, such as bleeding diatheses. Medications such as anti-coagulants were suspended prior to procedure in accordance to recommended guidelines^19^.

All patients scheduled for EBUS-TBNA procedure were considered for additional TBMC procedure under the jurisdiction of the managing physician. Notably, there were no strict selection criteria for TBMC procedure in our study, TBMC were considered in the following situations, namely: when uncommon tumours or benign disorders were suspected prior to the procedure or when specific immune-histochemical or molecular analyses were considered necessary.

TBMC was generally avoided on necrotic or cystic lymph nodes or when purulent materials were aspirated on TBNA, as they may suggest a higher pre-test probability of infection, especially tuberculosis in our region of practice. For TBMC, care was taken to avoid puncturing any target with abnormal overlying bronchial mucosa to mitigate the theoretical risk of poor wound healing, potentially leading to fistula formation.

#### Sedation and airway access

EBUS procedures were conducted by consultant pulmonologists with expertise in flexible and rigid bronchoscopy. Flexible bronchoscopy was first performed under conscious sedation or total intravenous anaesthesia in accordance with individual unit protocols. The decision for advanced airways (*i.e., rigid bronchoscope, endotracheal intubation, laryngeal mask airway*) was made at discretion of the bronchoscopist, considering the risks and benefits of the procedure for the patient^20^.

Various flexible echo-bronchoscopes (*EB1970UK, Pentax Medical, Japan; BF-UC180F, BF-UC190F, Olympus Medical, Japan; EB530US, Fujifilm, Japan*) were utilized for the procedures. Systematic examination of mediastinal and hilar lymph nodes was performed after endobronchial abnormalities were excluded during initial airway inspection by flexible bronchoscopy^1,3^. Each lymph node station was assessed, and their parameters (*size, shape, border, central hilar structure, vascularity on Doppler assessment*) were recorded.

#### EBUS-TBNA procedure

TBNA was conducted using needles of various sizes from 19 to 25 gauge (*ViziShot 2, ViziShot FLEX, Olympus Medical, Japan; Echotip*^*®*^* Ultra HD*^*™*^*, ProCore*^*™*^*, Cook Medical, Inc. USA*) determined by bronchoscopist discretion following standard procedural steps^1^. A minimum of three aspirations were performed with or without suction. Efforts were made to puncture through the same nodal entry point during each TBNA whenever possible to facilitate formation of a needle track for TBMC later. Pathologist-led rapid onsite cytology evaluation (ROSE) was routinely available only in QEH. The aspirated material was then transferred to a liquid-based cell-block for cytological analysis. In some cases, TBMC were performed upfront without TBNA.

#### EBUS-TBMC procedure

As mentioned, the target lesion was deliberately punctured with a TBNA needle multiple times at the exact same spot to facilitate creation of a needle tract, enabling easy insertion of a cryoprobe into the target lesion during TBMC later. An alternative approach was done using a 1.9mm high-frequency needle-knife (*KD-31C-1, Olympus*, *Japan*) to create a mucosal defect at the puncture site. Subsequently, the 1.1mm flexible ultrathin cryoprobe (*ERBE Medizintechnik, Tübingen, Germany*) was inserted through the working channel of the echobronchoscope, positioned at the mucosal defect, and directed into the core of the target lesion.

Once the cryoprobe was confirmed to be in the desired position on EBUS, *Doppler* ultrasound was activated to ensure that there were no significant surrounding vessels. The cryoprobe was then activated, and the echobronchoscope-cryoprobe assembly was removed *en-bloc* from the patient's natural or artificial airway. Upon successful specimen retrieval, the cryo-specimen was thawed in normal saline and immediately fixed in formalin for subsequent histological analysis. The freezing time and number of passes were not fixed and were adjusted based on the retrieved specimen. Generally, an incremental freezing time starting from 3–4 s was employed, and the procedure was repeated until the obtained specimen was deemed adequate by the bronchoscopist, barring any complications.

#### Post-procedural care

After the procedure, a routine chest radiograph was conducted to assess for the presence of pneumothorax and pneumomediastinum. Before discharging the patient, specific inquiries were made to detect potential signs and symptoms of vocal cord injury or mediastinitis. Prophylactic antibiotics were not routinely prescribed post-procedure. Patients were typically discharged 24 h after procedure with follow-up clinic appointments arranged in 2 weeks. We did not routinely perform repeat surveillance bronchoscopy unless clinically indicated or in the presence of suspected procedural complications, such as pneumothorax, pneumomediastinum, infection, etc.

### Definition

#### Diagnostic yield

Overall EBUS procedure was deemed conclusive if the specimens provided a formal cytological or histopathological diagnosis (*pathologically confirmed malignancy or specific benign disease*). The presence of a preponderance of small mature lymphocytes in EBUS-TBNA smears or cell block and/or lymphoid tissue in EBUS-TBMC specimen were considered representative sampling and deemed diagnostic only after at least six months of radiological surveillance which confirmed clinical stability^16–18,21^.

#### Freezing time and specimen size

The total freezing time for each TBMC procedure was recorded. The average freezing time per cryo-pass was then calculated by dividing the total freezing time by the number of cryo-passes. A representative case and calculation were illustrated in Supplementary Material. The specimen size in total aggregate diameter was calculated summing each largest diameter of the any single sample measured under the microscope.

#### Common malignancy vs. uncommon tumours and benign disorders

For the purposes of the study, all non-small cell lung carcinoma, small cell carcinoma, and metastatic carcinoma, were classified as common malignancy. Any other diagnosis was classified as uncommon tumours (*i.e., lymphoproliferative malignancy, germ cell tumour, thymic malignancy, *etc*.*) or benign disorders (*benign tumour or infective and inflammatory causes*)^16–18^.

### Statistical analysis

Data analysis was conducted using SPSS software (*version 20; Chicago, IL, USA*). The Shapiro–Wilk test was employed to assess normality. Normally distributed variables were presented as mean ± standard deviation (SD), while non-normally distributed variables were expressed as median and interquartile range (IQR). Categorical data were presented as absolute numbers and percentages, and comparisons were made using Pearson’s Chi-squared test or Fisher’s exact test. Independent sample t-tests and Mann–Whitney tests were utilized to compare normally and non-normally distributed variables between groups, respectively. A significance level of < 0.05 was applied.

### Ethical approval

The study protocol was approved by the medical research and ethics committee, Ministry of Health Malaysia, NMRR-ID-23-00,577-6XP (IIR).

## Results

**Figure 1:**
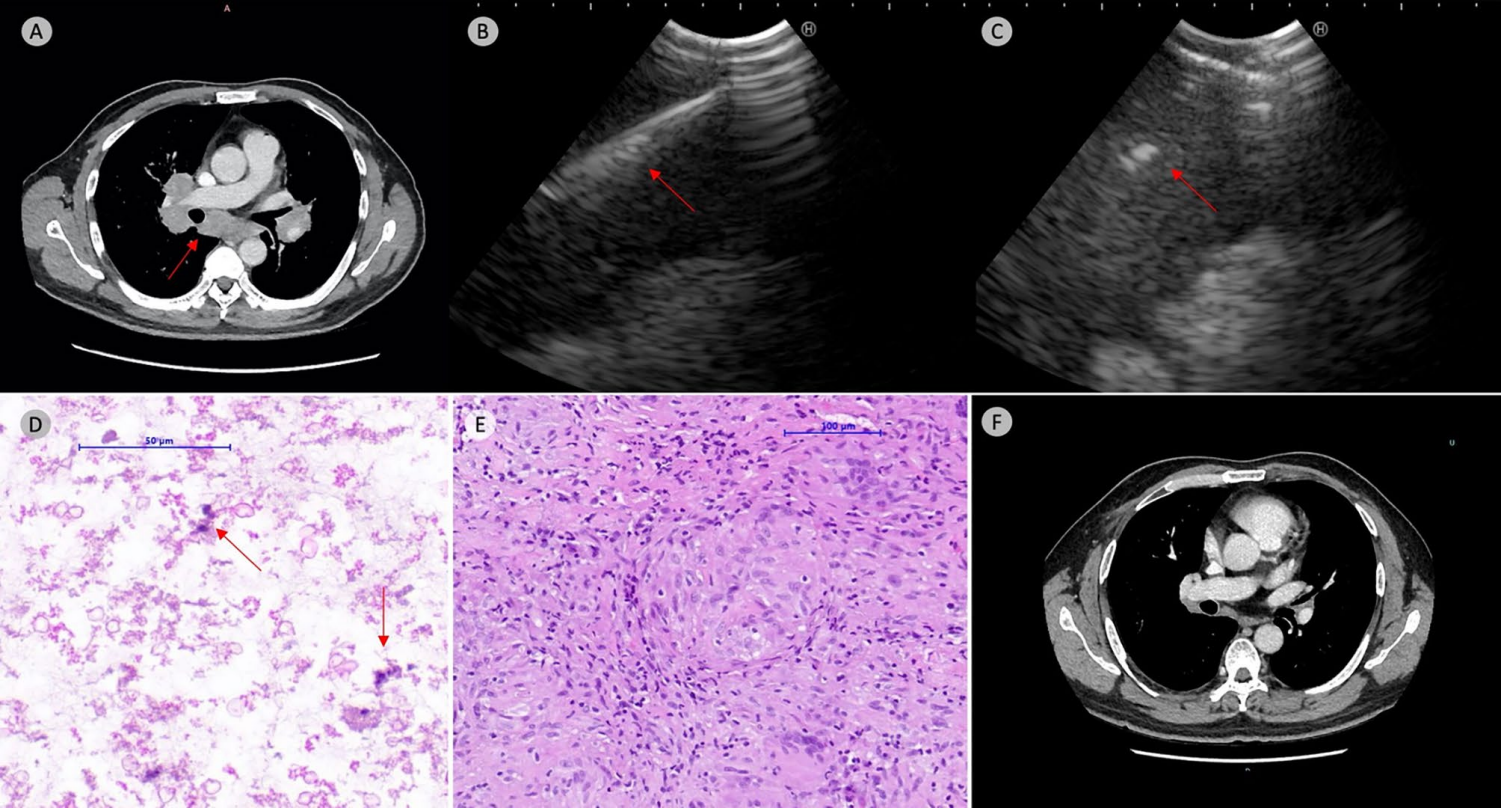
Representative case. Contrast enhanced computed tomography show bilateral hilar and mediastinal lymphadenopathy (*arrow*, *Panel A*). EBUS-TBNA was performed with 22-gauge TBNA needle with a total of 5 passes (*arrow, Panel B*). 1.1 mm cryoprobe was then inserted after mucosal incision with high-frequency needle knife (*arrow, Panel B*); a total of 5 cryo-passes was performed with total freezing time of 22 s (*average 4.4 s freezing time per cryo-pass*). TBNA cell block revealed only cellular debris with scanty macrophages (*arrow, hematoxylin & eosin stain, Panel D*). Collectively, the TBMC specimen measured 11 mm in total aggregate diameter which demonstrated a well-formed non-caseating granuloma consistent with clinical suspicion of sarcoidosis. (*hematoxylin & eosin stain, Panel E*). Post treatment with steroid and methotrexate demonstrate complete resolution of hilar and mediastinal lymphadenopathy (*Panel F*).

Throughout the study period, a total of 222 EBUS procedures were performed across all sites. TBMC was attempted in 137 cases (61.7%). Among these cases, the cryoprobe failed to be inserted in 8 cases (5.8%), resulting in an overall technical feasibility of 94.2% (Supplementary Table 1). In these eight cases, the blunt cryoprobe failed to be inserted into the target despite using a high-frequency needle knife and TBNA needle due to the tough and hard capsular wall of the lymph nodes (Supplementary Table 2). Consequently, a total of 129 EBUS-TBMC procedures were available for analysis. A representative case was shown in Fig. 1.

### Overall demographic and target lesions characteristic

The median age of our cohort was 63 (IQR 53–69) years, with two-thirds being male. Approximately half of the cases (51.2%) were performed on an outpatient basis. Concerning the target lesions, 87.6% were hilar or mediastinal lymph nodes, while 12.4% were peribronchial central lung masses. The median size for hilar or mediastinal lymph nodes was 17.0 (IQR 12.1–25.5) mm and for peribronchial central lung masses was 17.0 (IQR 11.0–37.3) mm. Lymph nodes were sampled in various stations, with the majority in station 4R (36.3%) and 7 (35.4%), followed by 11Rs/11Ri (15.9%). Complete characteristics were listed in Table 1.

**Table 1:** Overall demographic, target lesion and procedural characteristic.

Demographics & targets characteristic	
Median age (IQR), *years*	63 (53–69)
Gender, n (%)	
Male	78 (60.5)
Female	51 (39.5)
Ethnicity, n (%)	
Malay	35 (27.1)
Non-Malay Natives	37 (28.7)
Chinese	49 (38.0)
Indian	8 (6.2)
Admission, n (%)	
In-patient	63 (48.8)
Out-patient	66 (51.2)
Indication, n (%)	
Diagnostic	90 (69.8)
Staging	39 (30.2)
Target lesion, n (%)	
Lymph node	113 (87.6)
Central lung mass	16 (12.4)
Median target size (IQR), mm	
Lymph node	17.0 (12.1–25.5)
Central lung mass	17.0 (11.0–37.3)
Lymph node station, n (%)	
2R	1 (0.9)
4R	41 (36.3)
10R	4 (3.5)
11Rs/11Ri	18 (15.9)
2L	1 (0.9)
4L	3 (2.7)
11L	5 (4.4)
7	40 (35.4)
Procedural characteristic	
Median overall procedural time (IQR), min	55.0 (43.0–66.5)
Echobronchoscope, n (%)	
Fujifilm EB-530US	52 (40.3)
Pentax EB1970-UK	46 (35.7)
Olympus BF-UC180F	4 (3.1)
Olympus BF-UC190F	27 (20.9)
Airway access, n (%)	
Trans-oral	44 (34.1)
Laryngeal Mask Airway	69 (53.5)
Endotracheal Tube	11 (8.5)
Rigid Bronchoscopy	5 (3.9)
Rapid on-site examination, *n* (%)	5 (3.9)
Methods for initial cryobiopsy tract creation, n (%)	
Up-front high-frequency needle knife	8 (6.2)
19-gauge TBNA needle	13 (10.1)
21-gauge TBNA needle	18 (14.0)
22-gauge TBNA needle	87 (67.4)
25-gauge TBNA needle	3 (2.3)
Median TBNA pass (IQR), *passes*	4 (4–5)
Cryoprobe placement, n (%)	
After TBNA needle only	66 (51.2)
Needle knife up-front or assistance	63 (48.8)
Median cryo-pass (IQR), passes	4 (3–4)
Median total freezing time (IQR), seconds	24.0 (18.0–32.0)
Median average freezing time per pass (IQR), seconds	7.0 (5.5–9.3)
Overall diagnostic yield for EBUS, *n* (%)	114 (88.4)
Overall *short-term* complication, *n* (%)	20 (15.5)
Sedation related hypoventilation, *n* (%)	10 (7.8)
Pneumothorax, *n* (%)	0 (0.0)
Pneumomediastinum, *n* (%)	1 (0.8)
Bleeding, *n* (%)	9 (7.0)
Bleeding intervention, n	
Prolonged suction	5
Adrenaline-saline instillation	1
Switch bronchoscope	3

### Overall procedural characteristic and outcome

The overall median procedural time was 55.0 (IQR 43.0–66.5) minutes, measured from the administration of sedative medication to the conclusion of procedures. Advanced airway (*endotracheal intubation, rigid bronchoscopy*) accounted for 12.4%, supraglottic airway (*laryngeal mask airway*) for 53.5%, and trans-oral route for 34.1%. (Table 1).

Up-front TBMC were performed in 29/129 cases (22.5%) without prior TBNA. In these cases, cryo-biopsy tract was created using a high frequency needle knife and using the needle method in 8 and 21 cases respectively. Needle method was successful in all but 1 case, where cryo-biopsy tract was eventually created with a high frequency needle knife. For the remaining 100/129 cases, biopsy tract creation was attempted concurrently during TBNA, which was successful in 46% cases. The remaining 54 cases (54%) required needle knife for tract creation despite prior TBNA attempts. Overall, a conclusive diagnosis was established in 114 out of 129 cases (88.4%) in which TBMC was carried out. The histology of TBMC cases and their final outcome were listed in Supplementary Tables 3, 4 and5.

Remarkably, the cohort exhibited no recorded incidents of pneumothorax, with only one encounter of self-limiting pneumomediastinum (0.8%). Bleeding occurred in nine cases (7%) which was managed expectantly with prolonged suction (n = 5), changing to therapeutic bronchoscope for more effective suction (n = 3) and adrenaline saline instillation (n = 1).

### Correlation of TBMC specimen size to overall diagnostic yield

**Figure 2:**
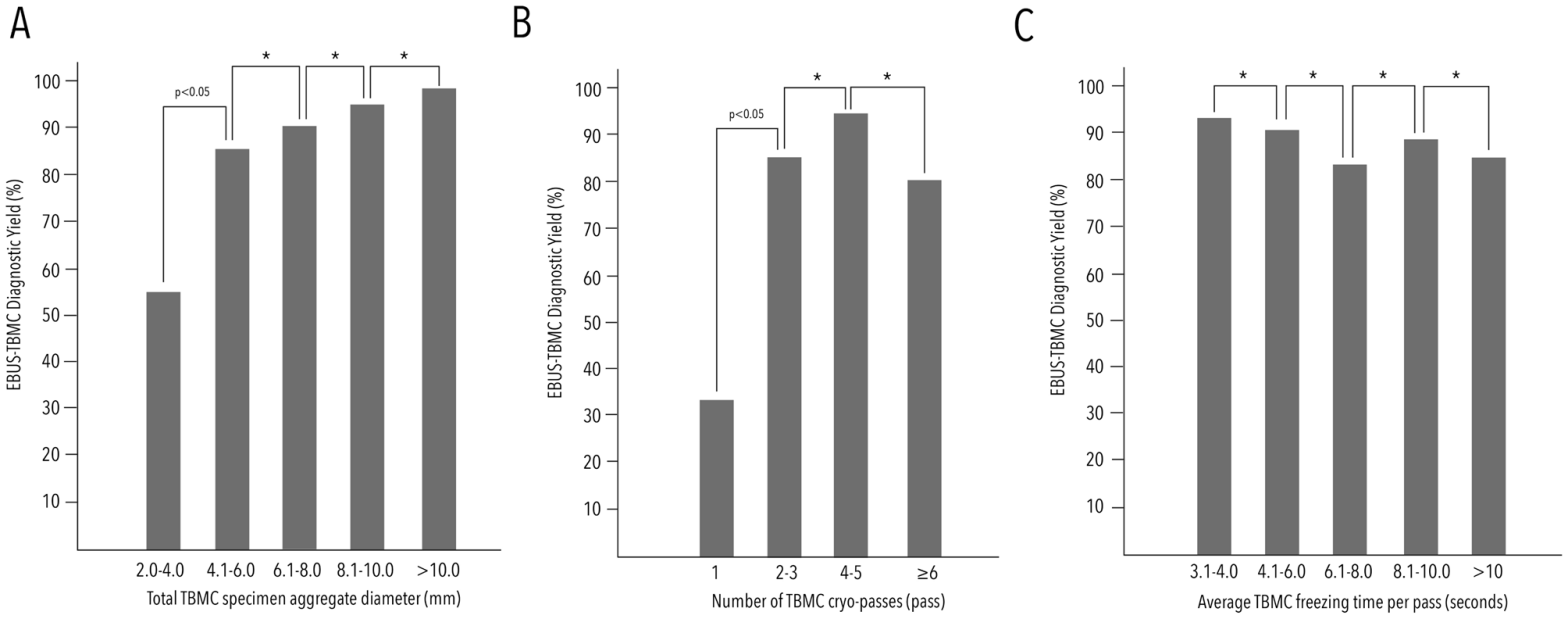
Difference of EBUS-TBMC diagnostic yield across different TBMC specimen sizes in total aggregate diameter (Panel A), cryo-passes (Panel B) and average freezing time per cryo-pass (Panel C). * signify *p* > 0.05.

The overall median total aggregate diameter of TBMC specimens was 7.0 (IQR 5.0–9.5) mm. Conclusive TBMC procedures were associated with a larger specimen size [7.0 (IQR 5.0–10.0) vs. 5.0 (IQR 4.0–7.0) mm, *p* < 0.01]. TBMC demonstrated a significant increment in diagnostic yield (r = + 0.240, *p* < 0.01) up to a cut-off specimen size of 4.1–6.0 mm. Beyond this threshold, specimens exceeding 6 mm did not exhibit a discernible variance in the diagnostic yield. (Fig. 2, Table 2, Supplementary Table 6).

**Table 2:** Diagnostic yield of EBUS-TBMC at different specimen size thresholds.

Total aggregate diameter of specimen, mm	Diagnostic yield, n/N (%)	*p*-value*
2.0–4.0	5/9 (55.6)	–
4.1–6.0	45/52 (86.5)	0.026
6.1–8.0	31/34 (91.2)	0.512
8.1–10.0	24/25 (96.0)	0.466
> 10.1	9/9 (100.0)	0.543

*p-value in comparison to previous specimen size threshold.

### Correlation of TBMC cryo-passes to specimen size and overall diagnostic yield

Overall, a median of 4 (IQR 3–4) cryo-passes were performed in our cohort. A weak but significant positive correlation was observed between number of cryo-passes and the specimen size (*r* = + 0.240, *p* < 0.01), as well as the overall diagnostic yield (*r* = + 0.196, *p* < 0.05).

Conclusive TBMC procedures exhibited a higher median cryo-pass compared to inconclusive procedures [4 (IQR 3–4) vs. 3 (IQR 2–4) passes, *p* < 0.05]. Additionally, the diagnostic yield of 2–3 cryo-passes was significantly higher compared to a single pass of TBMC (85.0% vs. 33.3%, *p* < 0.05). Although 4–5 passes demonstrated a higher diagnostic yield at 95.1%, this did not reach statistical significance compared to 2–3 cryo-passes. (Fig. 2, Table 3, Supplementary Table 7).

**Table 3:** Diagnostic yield of EBUS-TBMC at different cryo-passes ranges.

Cryo-passes, *pass*	Diagnostic yield, n/N (%)	*p*-value*
1 pass	1/3 (33.3)	–
2–3 pass	51/60 (85.0)	0.021
4–5 pass	58/61 (95.1)	0.065
6 pass or more	4/5 (80.0)	0.174

*p-value in comparison to previous cryo-passes range.

### Correlation of TBMC freezing time to specimen size to overall diagnostic yield

The median total freezing time was 24.0 (IQR 18.0–32.0) seconds per case, with an average freezing time per cryo-pass at 7.0 (IQR 5.5–9.3) seconds per case. Interestingly, a non-statistically significant negative correlation was observed between specimen size (*r* = − 0.040, *p* > 0.05) and diagnostic yield (*r* = − 0.052, *p* > 0.05) with longer freezing times. (Supplementary Table 8).

The median freezing time did not show a significant difference between conclusive and inconclusive TBMC cases [7.0 (IQR 5.5–8.8) vs. 8.0 (IQR 6.0–10.0) seconds, *p* > 0.05]. The highest diagnostic yield were recorded in the 3.1 to 4.0 s freezing time group. Exploration of various freezing time thresholds revealed no significant differences in diagnostic yield (Table 4).

**Table 4:** Diagnostic yield EBUS-TBMC at different freezing time ranges.

Freezing time, *seconds*	Diagnostic yield, *n/N* (%)	*p*-value*
3.1–4.0	94.4, 17/18	–
4.1–6.0	91.2, 31/34	0.674
6.1–8.0	83.3, 30/36	0.327
8.1–10.0	89.3, 25/28	0.497
> 10.1	84.6, 11/13	0.671

*p-value in comparison to previous freezing time range.

### Subgroup analysis comparing the diagnostic yield of TBNA to TBMC in different diagnosis groups

Overall, the diagnostic yield of TBMC was 88.4% (114/129) compared to 53% (53/100) for TBNA. EBUS exhibited a significantly higher diagnostic yield in cases of common malignancies compared to uncommon tumors and benign disorders (96.1% vs. 85.4%, *p* < 0.05). Within the realm of common malignancies, the diagnostic yields of TBNA and TBMC were statistically comparable (60.0% vs. 96.4%, *p* = 0.078). However, a notable disparity emerged in cases of uncommon tumors and benign disorders, where TBMC outperformed TBNA (48.8% vs. 82.9%, *p* < 0.01). (Supplementary Table 9, 10, 11 and 12).

## Discussion

Cryobiopsy in respiratory endoscopy has been in practice for almost a decade, experiencing a resurgence in popularity, particularly in the diagnosis of interstitial lung disease using TBLC (ILD-TBLC)^22,23^. The technical intricacies of ILD-TBLC have been extensively explored and tested in animal studies, covering aspects such as the number of passes, freezing time, and their correlation with specimen size and quality; including the utilization of newer-generation single-use ultrathin flexible cryoprobes^24–26^. Thus, specific expert recommendations on the number of passes and freezing time for ILD-TBLC are well-established^27^. Similarly, recommendations exist for transbronchial cryobiopsy of peripheral pulmonary lesions (PPL-TBLC)^28,29^. On the other hand, technical data for EBUS-TBMC is currently lacking, potentially due to the challenges of creating sizeable mediastinal lymph nodes in animal models for testing^30^. Given the initial scarcity of technical details, various number of cryo-passes and freezing times had been explored in our cohort which enables the exploratory analysis of this novel procedure.

Firstly, in the context of ILD-TBLC, a gross specimen size of at least 5 mm is suggested to be optimal for histopathologic analysis^27^. However, the optimum specimen size for TBMC remains unknown. Existing literature reports a range of specimen sizes from 3.8 to 4.6 mm with variations in cryo-passes and freezing time^16,17,31^. In our study, we observed a positive correlation between specimen size and diagnostic yield, which plateaued at 4.1 to 6.0 mm of total aggregate specimen size. We hypothesized that the plateau of diagnostic yield is likely due to the intranodal heterogeneity frequently observed in pathological lymph nodes^32^. As TBMC often samples only a limited region of the target lesion, the actual biopsy site may not be wholly representative despite providing a larger specimen size. Similarly, in ILD-TBLC using the ultrathin cryoprobe in an animal model, an increase in specimen size did not consistently impact the representativeness of specimens^25^. Further studies exploring this intriguing finding in the future could provide valuable insights into optimizing the diagnostic process in TBMC procedures, especially in utilizing advanced imaging tools such as elastography to guide the biopsy site.

Secondly, the optimal number of TBMC cryo-passes remains unknown. In TBNA, it is recommended to perform at least three needle passes and, even more passes if molecular analysis is required^33^. In the TBMC literature, 2–4 passes were generally shown to achieve a good diagnostic yield^14,16,31,34^. Fan et al*.*^17^ had also demonstrated that just one additional TBMC pass can significantly complement the diagnostic performance of TBNA. Importantly, our study had revealed a significant positive correlation between the number of cryo-passes and specimen size. Our exploratory data further indicate that diagnostic yield plateaued after 2–3 cryo-passes. This finding is crucial, especially in cases performed through the trans-oral route or supra-glottic airway, where unnecessary cryo-passes should be avoided to limit potential vocal cord irritation and patient discomfort.

Thirdly, while freezing times have been extensively explored in ILD-TBLC and PPL, this aspect has not received thorough investigation in TBMC^23,27,29^. The first TBMC report in humans initiated with a freezing time of 15 s, later reduced to 7 s in a randomized controlled trial by the same group^11,16^. Subsequent studies have reported various freezing times ranging from 3 to 7 s ^14,16–18,31,34^. One might intuitively assume that longer freezing times would yield larger specimens, akin to the observations in ILD-TBLC. However, the fundamental difference is that TBLC specimens are retrieved through normal airway conduit, while TBMC specimens are obtained through an artificially created airway mucosal defect. In an animal study, utilizing an ultrathin cryoprobe with an outer sheath in ILD-TBLC, a maximum freezing time of 6–10 s was demonstrated. Beyond this range, specimens failed to be retrieved due to sheath compression^24,25^. Aligning with this, our data showed a non-statistically significant negative correlation between freezing time and specimen size. This suggested that the artificially created mucosal defect and track might not be large enough to retrieve larger TBMC samples, especially with longer freezing times. However, it is crucial to note that the freezing time in our study was not executed in a pre-defined incremental or decremental protocol; instead, an average freezing time per pass was calculated from the total activation time and cryo-passes. Therefore, this finding should be interpreted with caution. Nonetheless, we believe this pave the way for future studies to confirm and further explore this important aspect of TBMC procedures.

As a secondary endpoint of this explorative analysis, the overall diagnostic yield of TBMC was 88.4% in our cohort, in line with various other recent studies quoting diagnostic yields of 71.7–96.0%^13,16–18,34^. Importantly, our findings reaffirmed the superior role of TBMC in the diagnosis of uncommon tumours and benign disorders compared to TBNA in a *real-world* setting^16–18^. Our study also reinforced TBMC as a safe procedure, with only 0.7% incidence of self-limiting pneumomediastinum, consistent with literature at 0.5–1.0%^16,17^. The clinically significant bleeding rate in our cohort was 7%, which was consistent with TBMC literature, ranging from 6.4 to 14.0%, despite our various cryo-passes and freezing times^16–18^. Overall, the bleeding rate of TBMC in literature seemed to be lower compared to the bleeding rate of transbronchial lung cryobiopsy (TBLC), which was around 29.3% in meta-analysis. Moreover, no severe life-threatening bleeding complications have been reported yet in the TBMC literature compared to the TBLC literature^28,35^. Thus, more data on the TBMC safety profile is essential to ascertain the actual bleeding risk of this novel procedure and to determine the need for additional bleeding mitigation methods as in TBLC. Interestingly, we also demonstrated that TBMC is not always feasible in all cases in a *real-world* setting, as the cryoprobe failed to be inserted in eight cases due to a tough and dense lymph node capsule in our cohort. This is likely due to the blunt nature of the cryoprobe, which does not allow effective penetration of an occasionally stony-hard metastatic lymph node.

This study had several important limitations.

Firstly, the retrospective design and the absence of a standardized procedural protocol indicate that the actual causal relationship between the number of cryo-passes and freezing time to diagnostic yield cannot be determined confidently. However, we believed that the heterogeneity in procedural characteristics mirrors *real-world* clinical practice, making the findings relevant for clinicians in the region. Moreover, the unblinded study design may have introduced review bias favoring TBMC over TBNA from the pathologist's perspective, resulting in a significantly lower TBNA yield (53%) in our cohort. This bias was also demonstrated by Poletti et al*.*^15^, who found that in an unblinded cohort, pathologists tended to prefer TBMC material over TBNA specimens, especially for molecular analysis. We also believed that this bias was more apparent in our region, where cytopathology services were less developed^6^.

Another limitation of our study was the absence of ROSE in the majority of our cases. Although ROSE had been shown to complement bronchoscopic procedures by reducing procedural time and the need for additional procedures; its role in increasing the diagnostic yield of EBUS-TBNA has not been proven^33,36^. Nevertheless, a recent study demonstrated that using ROSE to stratify and select patients undergoing EBUS-TBNA for additional TBMC procedures resulted in an additional 43.7% diagnostic yield to overall EBUS procedures^34^. Thus, implementing an algorithm for case selection in TBMC based on intra-procedural ROSE during TBNA may prove to be a valuable strategy.

Thirdly, our study recorded a prolonged procedural time due to the inclusion of time required for anesthetic initiation, placement of the artificial airway, and initial airway examination. Additionally, we did not separately record the timings for TBNA and TBMC. Previous studies had indicated that overall EBUS procedural duration typically range from 31.9 to 33.9 min, with TBMC specifically requiring an additional 2.3–5.3 min^16,17,31^. Future studies that provide a more detailed breakdown of procedure times for various cryo-passes and freezing times would offer a more precise evaluation of procedural efficiency.

Next, the follow-up duration in our study may not be optimal. As of the time of writing, the current CT surveillance period for the 15 conclusive TBMC cases with lymphoid tissue was less than 12 months. Consequently, there is a possibility that the overall diagnostic yield could be overestimated. Reassuringly, most literature established a 6-month radiological surveillance period to define the stability of hilar and mediastinal lymphadenopathy^16–18,21^. The limited follow-up duration also meant that the long-term complications remained unknown. Notably, as of the 6-month follow-up for this cohort of patients, no delayed complications were encountered.

Lastly, our study only exclusively explored the technical details of the 1.1 mm cryoprobe in TBMC procedures. Recent reports using the larger 1.7 mm cryoprobe for TBMC had shown promising results with no increments in complication rates^37,38^. Interestingly, a recent report on EBUS-IFB using conventional 1.8 mm forceps also demonstrated procedural feasibility, achieving a high diagnostic yield of 99.5%^39^. However, a recent randomized controlled trial comparing EBUS-TBNA with supplementary IFBs and TBMC showed that although both biopsy techniques enhanced the diagnostic yield of EBUS-TBNA, TBMC was able to retrieve larger specimens with more preserved cellular architecture and fewer crushed artifacts, making it more suitable for lung cancer molecular testing^18^. Thus, further comparative studies using different sizes of cryoprobes and biopsy techniques regarding diagnostic yield, suitability for molecular analysis, and cost-effectiveness are eagerly anticipated.

## Conclusion

EBUS-TBMC using the ultrathin cryoprobe is useful and safe in the diagnosis of mediastinal and hilar pathologies. The diagnostic yield plateaued once the total aggregate specimen size reached 4.1–6.0 mm. Optimal performance is linked to 2–3 cryo-passes and a freezing time of 3.1–4.0 s. Prospective studies are necessary to validate these findings.

### Supplementary Information

Supplementary Information.

## Data Availability

The data that support the findings of this study are available from the corresponding author, SSK, upon reasonable request.

## Abbreviations

EBUS: Endobronchial ultrasound
IFB: Intranodal forceps biopsy
ILD: Interstitial lung disease
IQR: Interquartile range
PPL: Peripheral pulmonary lesion
SD: Standard deviation
TBLC: Transbronchial lung cryobiopsy
TBMC: Transbronchial mediastinal cryobiopsy
TBNA: Transbronchial needle aspiration

## References

[1] Yasufuku, K. *et al.* A prospective controlled trial of endobronchial ultrasound-guided transbronchial needle aspiration compared with mediastinoscopy for mediastinal lymph node staging of lung cancer. *J. Thorac. Cardiovasc. Surg.* **142**(6), 1393–400.e1 (2011). 21963329 10.1016/j.jtcvs.2011.08.037

[2] Gompelmann, D., Eberhardt, R. & Herth, F. J. Endobronchial ultrasound. *Endosc. Ultrasound* **1**(2), 69–74 (2012). 24949340 10.7178/eus.02.003 PMC4062211

[3] Vilmann, P. *et al.* Combined endobronchial and oesophageal endosonography for the diagnosis and staging of lung cancer. European Society of Gastrointestinal Endoscopy (ESGE) Guideline, in cooperation with the European Respiratory Society (ERS) and the European Society of Thoracic Surgeons (ESTS). *Eur. Respir. J.* **46**(1), 40–60 (2015). 26034128 10.1183/09031936.00064515

[4] Silvestri, G. A. *et al.* Methods for staging non-small cell lung cancer: Diagnosis and management of lung cancer, 3rd ed: American College of Chest Physicians evidence-based clinical practice guidelines. *Chest* **143**(5 Suppl), e211S-e250S (2013). 23649440 10.1378/chest.12-2355

[5] Labarca, G. *et al.* Diagnostic accuracy of endobronchial ultrasound transbronchial needle aspiration in lymphoma. A systematic review and meta-analysis. *Ann. Am. Thorac. Soc.* **16**(11), 1432–1439 (2019). 31291126 10.1513/AnnalsATS.201902-175OC

[6] Kho, S. S., Chan, S. K., Ismail, A. M. & Tie, S. T. Tissue-clot-coagulum cell block in EBUS/TBNA specimen adequacy: A real world experience. *Diagn Cytopathol.* **50**(12), 583–585 (2022). 36135808 10.1002/dc.25056

[7] Lindeman, N. I. *et al.* Updated molecular testing guideline for the selection of lung cancer patients for treatment with targeted tyrosine kinase inhibitors: Guideline from the college of American pathologists, the international association for the study of lung cancer, and the association for molecular pathology. *J. Mol. Diagn.* **20**(2), 129–159 (2018). 29398453 10.1016/j.jmoldx.2017.11.004

[8] Minami, D. *et al.* Comparing the clinical performance of the new 19-G ViziShot FLEX and 21- or 22-G ViziShot 2 endobronchial ultrasound-guided transbronchial needle aspiration needles. *Intern. Med.* **57**(24), 3515–3520 (2018). 30146572 10.2169/internalmedicine.0967-18 PMC6355416

[9] Herth, F. J., Morgan, R. K., Eberhardt, R. & Ernst, A. Endobronchial ultrasound-guided miniforceps biopsy in the biopsy of subcarinal masses in patients with low likelihood of non-small cell lung cancer. *Ann. Thorac. Surg.* **85**(6), 1874–1878 (2008). 18498786 10.1016/j.athoracsur.2008.02.031

[10] Chrissian, A., Misselhorn, D. & Chen, A. Endobronchial-ultrasound guided miniforceps biopsy of mediastinal and hilar lesions. *Ann. Thorac. Surg.* **92**(1), 284–288 (2011). 21718857 10.1016/j.athoracsur.2011.03.069

[11] Zhang, J. *et al.* Primary mediastinal seminoma achieved by transbronchial mediastinal cryobiopsy. *Respiration* **99**(5), 426–430 (2020). 32050197 10.1159/000505936

[12] Genova, C. *et al.* Potential application of cryobiopsy for histo-molecular characterization of mediastinal lymph nodes in patients with thoracic malignancies: A case presentation series and implications for future developments. *BMC Pulm. Med.* **22**(1), 5 (2022). 34996404 10.1186/s12890-021-01814-x PMC8741535

[13] Ariza-Prota, M. A. *et al.* Transbronchial mediastinal cryobiopsy in the diagnosis of mediastinal lymph nodes: A case series: How to do it. *Arch Bronconeumol* **58**(10), 718–721 (2022). 35697564 10.1016/j.arbres.2022.05.006

[14] Gonuguntla, H. K. *et al.* Endobronchial ultrasound-guided transbronchial cryo-nodal biopsy: A novel approach for mediastinal lymph node sampling. *Respirol. Case Rep.* **9**(8), e00808 (2021). 34262775 10.1002/rcr2.808 PMC8264746

[15] Poletti, V. *et al.* EBUS-guided cryobiopsy in the diagnosis of thoracic disorders. *Pulmonology* **S2531–0437**(23), 00223–00224 (2024). 10.1016/j.pulmoe.2023.11.008 38182468

[16] Zhang, J. *et al.* Transbronchial mediastinal cryobiopsy in the diagnosis of mediastinal lesions: A randomised trial. *Eur. Respir. J.* **58**(6), 2100055 (2021). 33958432 10.1183/13993003.00055-2021

[17] Fan, Y. *et al.* Transbronchial needle aspiration combined with cryobiopsy in the diagnosis of mediastinal diseases: A multicentre, open-label, randomised trial. *Lancet Respir. Med.* **S2213–2600**(22), 00392–00397 (2022). 10.1016/S2213-2600(22)00392-7 36279880

[18] Cheng, T. L. *et al.* Comparison of cryobiopsy and forceps biopsy for the diagnosis of mediastinal lesions: A randomised clinical trial. *Pulmonology* **S2531–0437**(23), 00240–00244 (2024). 10.1016/j.pulmoe.2023.12.002 38182469

[19] Abuqayyas, S., Raju, S., Bartholomew, J. R., Abu Hweij, R. & Mehta, A. C. Management of antithrombotic agents in patients undergoing flexible bronchoscopy. *Eur. Respir. Rev.* **26**(145), 170001 (2017). 28724561 10.1183/16000617.0001-2017 PMC9488780

[20] Kho, S. S., Soo, C. I., Nasaruddin, M. Z., Ngan, K. W. & Abdul Rahaman, J. A. Multimodal linear endobronchial ultrasound guided mediastinal lymph node biopsy in the diagnosis of isolated mediastinal lymphadenopathy. *Proc. Singap. Healthc.* **31**, 20101058221111656 (2022). 10.1177/20101058221111655

[21] Munden, R. F. *et al.* Managing incidental findings on thoracic CT: Mediastinal and cardiovascular findings. A white paper of the ACR incidental findings committee. *J. Am. Coll. Radiol.* **15**(8), 1087–96 (2018). 29941240 10.1016/j.jacr.2018.04.029

[22] Babiak, A. *et al.* Transbronchial cryobiopsy: A new tool for lung biopsies. *Respiration* **78**(2), 203–208 (2009). 19246874 10.1159/000203987

[23] Ravaglia, C. *et al.* Diagnostic yield and risk/benefit analysis of trans-bronchial lung cryobiopsy in diffuse parenchymal lung diseases: A large cohort of 699 patients. *BMC Pulm Med.* **19**(1), 16 (2019). 30651103 10.1186/s12890-019-0780-3 PMC6335717

[24] Hetzel, J., Linzenbold, W., Boesmueller, H., Enderle, M. & Poletti, V. Evaluation of efficacy of a new cryoprobe for transbronchial cryobiopsy: A randomized. *Controll. Vivo Anim. Study Respir.* **99**(3), 248–256 (2020). 10.1159/000506017 32101862

[25] Yarmus, L. B. *et al.* A randomized controlled trial of a novel sheath cryoprobe for bronchoscopic lung biopsy in a porcine model. *Chest* **150**(2), 329–336 (2016). 26836935 10.1016/j.chest.2016.01.018

[26] Franke, K. J. *et al.* A new tool for transbronchial cryobiopsies in the lung: An experimental feasibility ex vivo study. *Respiration* **91**(3), 228–234 (2016). 26901791 10.1159/000443990

[27] Hetzel, J. *et al.* Transbronchial cryobiopsies for the diagnosis of diffuse parenchymal lung diseases: Expert statement from the cryobiopsy working group on safety and utility and a call for standardization of the procedure. *Respiration* **95**(3), 188–200 (2018). 29316560 10.1159/000484055

[28] Tang, Y. *et al.* Transbronchial lung cryobiopsy for peripheral pulmonary lesions. *A Narrat. Rev. Pulmonol.* **S2531–0437**(23), 00163 (2023). 10.1016/j.pulmoe.2023.08.010 37914556

[29] Kho, S. S. *et al.* Exploring the optimal freeze time and passes of the ultrathin cryoprobe in transbronchial cryobiopsy of peripheral pulmonary lesions. *ERJ Open Res.* **10**(1), 00506–02023 (2024). 38259810 10.1183/23120541.00506-2023 PMC10801766

[30] Franke, K. J. *et al.* The cryo-needle: A new tool for histological biopsies. *A Feasibility Study Lung* **191**(6), 611–617 (2013). 23990134 10.1007/s00408-013-9502-4

[31] Ariza-Prota, M. *et al.* Endobronchial ultrasound-guided transbronchial mediastinal cryobiopsy in the diagnosis of mediastinal lesions: safety, feasibility and diagnostic yield: Experience in 50 cases. *ERJ Open Res.* **9**(2), 00448–02022 (2023). 37077551 10.1183/23120541.00448-2022 PMC10107076

[32] Kurimoto, N. *et al.* Targeting area in metastatic lymph nodes in lung cancer for endobronchial ultrasonography guided transbronchial needle aspiration. *J. Bronchol.* **15**, 134–213 (2008). 10.1097/LBR.0b013e31817ec366

[33] Wahidi, M. M. *et al.* Technical aspects of endobronchial ultrasound-guided transbronchial needle aspiration: CHEST guideline and expert panel report. *Chest* **149**(3), 816–835 (2016). 26402427 10.1378/chest.15-1216

[34] Maturu, V. N., Prasad, V. P., Vaddepally, C. R., Dommata, R. R. & Sethi, S. Endobronchial ultrasound-guided mediastinal lymph nodal cryobiopsy in patients with nondiagnostic/inadequate rapid on-site evaluation: A new step in the diagnostic algorithm. *J. Bronchol. Interv. Pulmonol.* **31**(1), 2–12 (2024). 10.1097/LBR.0000000000000913 36877194

[35] Sryma, P. B. *et al.* Efficacy of radial endobronchial ultrasound (R-EBUS) guided transbronchial cryobiopsy for peripheral pulmonary lesions (PPL): A systematic review and meta-analysis. *Pulmonology* **29**(1), 50–64 (2023). 33441246 10.1016/j.pulmoe.2020.12.006

[36] Huang, H. *et al.* Application of bronchoscopy in the diagnosis and treatment of peripheral pulmonary lesions in China: A national cross-sectional study. *J. Cancer* **14**(8), 1398–1406 (2023). 37283786 10.7150/jca.84220 PMC10240658

[37] Gershman, E., Amram Ikan, A., Pertzov, B., Rosengarten, D. & Kramer, M. R. Mediastinal “deep freeze”-transcarinal lymph node cryobiopsy. *Thorac. Cancer* **13**(11), 1592–1596 (2022). 35474417 10.1111/1759-7714.14422 PMC9161345

[38] Takemura, C. *et al.* Thoracic SMARCA4-deficient undifferentiated tumor diagnosed by transbronchial mediastinal cryobiopsy: A case report. *Thorac. Cancer* **14**(10), 953–957 (2023). 36828806 10.1111/1759-7714.14830 PMC10067353

[39] Konno-Yamamoto, A. *et al.* Feasibility of modified endobronchial ultrasound-guided intranodal forceps biopsy: A retrospective analysis. *Respiration* **102**(2), 143–153 (2023). 36543151 10.1159/000528644

