# Supplementary material for: An explorative analysis on the optimal cryo-passes and freezing time of the ultrathin cryoprobe in endobronchial ultrasound-guided transbronchial mediastinal cryobiopsy
Source: Sci Rep. 2024 Aug 12;14:18653. doi: 10.1038/s41598-024-69702-y (PMC11319585; doi:10.1038/s41598-024-69702-y)
Supplement: Supplementary file 1 — Supplementary Information. [file 41598_2024_69702_MOESM1_ESM.docx]

**SUPPLEMENTARY MATERIAL**

**An Explorative Analysis On The Optimal Cryo-Passes and Freezing Time of The Ultrathin Cryoprobe in Endobronchial Ultrasound-Guided Transbronchial Mediastinal Cryobiopsy**

Sze Shyang KHO^1^, Shirin Hui TAN^2^, Chun Ian SOO^3^, Hema Yamini Devi RAMARMUTY^4^, Chan Sin CHAI^1^, Nai Chien HUAN^4^, Khai Lip NG^4^, Yuji MATSUMOTO^5^, Venerino POLETTI^6,7^, Siew Teck TIE^1^

^1^Division of Respiratory Medicine, Department of Medicine, Sarawak General Hospital, Ministry of Health Malaysia, Kuching, Sarawak, Malaysia.

^2^Clinical Research Centre, Sarawak General Hospital, Institute for Clinical Research, National Institutes of Health, Ministry of Health Malaysia, Kuching, Sarawak Malaysia

^3^Division Of Respiratory Medicine, Department Of Medicine, University Malaya Medical Centre, University Malaya, Kuala Lumpur, Malaysia

^4^Department of Respiratory Medicine, Queen Elizabeth Hospital, Ministry of Health Malaysia, Kota Kinabalu, Sabah, Malaysia.

^5^Department of Endoscopy, Respiratory Endoscopy Division, National Cancer Center Hospital, Tokyo, Japan

^6^Department of Medical Specialities-Pulmonology, GB Morgagni Hospital, Forlì, Italy

^7^Department of Medical and Surgical Sciences (DIMEC), Bologna University, Bologna, Italy.

**Calculation of Average Freezing Time per Cryo-pass**

***Case example***

A total of 4 cryo-passes were performed for Subject-001 with

- 1^st^ cryo-pass 4 seconds
- 2^nd^ cryo-pass 8 seconds
- 3^rd^ cryo-pass 10 seconds
- 4^th^ cryo-pass 12 seconds

Hence, the total freezing time

= (4 + 8 + 10 + 12) seconds

= 34 seconds

Hence, the average freezing time per cryo-pass

= 34 seconds / Total number of cryo-passes

= 34 seconds / 4

= 8.50 seconds


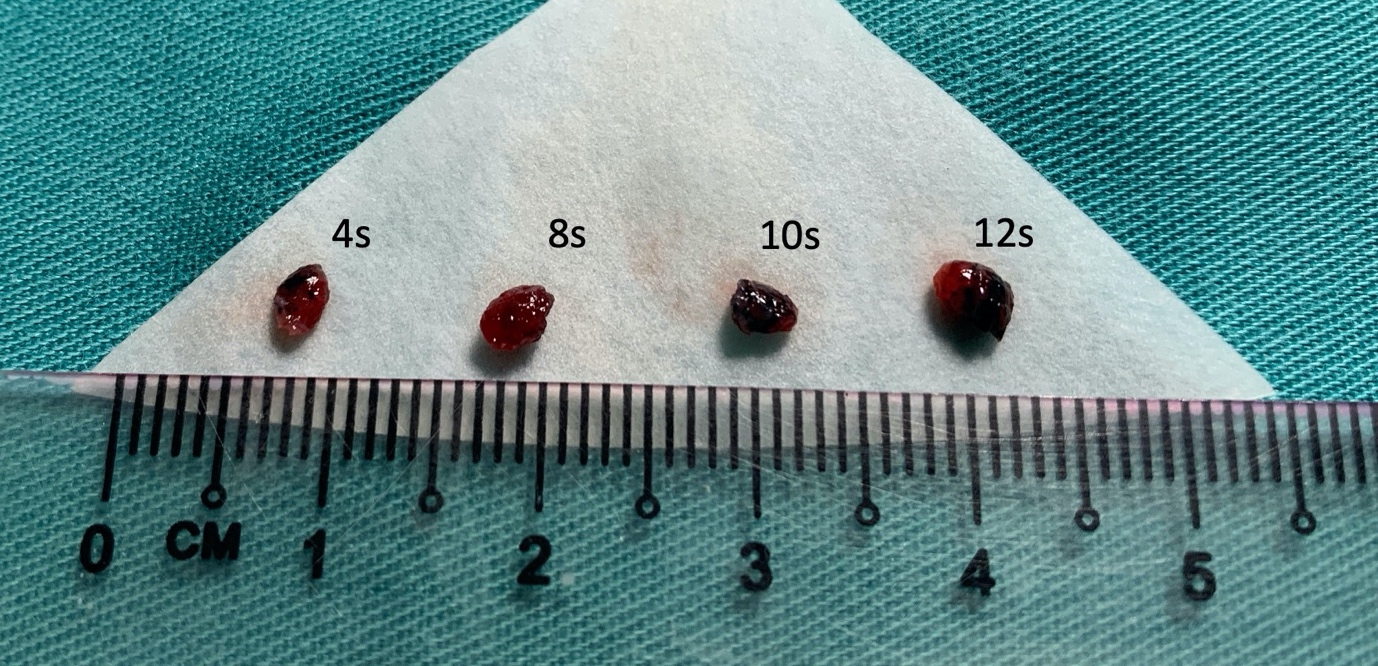


***Supplementary Figure 1.0***  Gross specimens from the 1.1mm cryoprobe with different freezing times

| **Supplementary Table 1 EBUS-TBMC cases distribution among different sites** | | | | |
| --- | --- | --- | --- | --- |
| ***Site*** | ***SGH*** | ***UMMC*** | ***QEH*** | ***Total*** |
| Total EBUS performed*, n* | 132 | 60 | 30* | 222 |
| Total EBUS-TBMC attempted, *n (%)* | 80 (60.6) | 53 (88.3) | 4 (13.3) | 137 (61.7) |
| Failed cryoprobe insertion. *n (%)* | 7 (8.75) | 1 (1.89) | 0 (0.0) | 8 (5.8) |
| Included for analysis, *n* | 73 | 52 | 4 | **129** |

**EBUS-TBMC service started only in January 2023*

| **Supplementary Table 2 Reason for failed cryoprobe insertion and final diagnosis** | | |
| --- | --- | --- |
| ***Case*** | ***Reason for failed probe insertion*** | ***Final diagnosis via EBUS-TBNA*** |
| 1 | Tough capsular wall | Metastatic adenocarcinoma – breast |
| 2 | Tough capsular wall | Lymphoid tissue |
| 3 | Tough capsular wall | Metastatic nasopharyngeal carcinoma |
| 4 | Tough capsular wall | Lymphoid tissue |
| 5 | Tough capsular wall | Blood and degenerated cells (inconclusive) |
| 6 | Tough capsular wall | Adenocarcinoma lung |
| 7 | Tough capsular wall | Metastatic adenocarcinoma lung |
| 8 | Tough capsular wall and patient unable to tolerate procedure further due to intractable cough* | Metastatic adenocarcinoma lung |

**procedure performed under conscious sedation*

| **Supplementary Table 3 Histology of conclusive EBUS-TBMC cohort (N=114)** | | | | | |
| --- | --- | --- | --- | --- | --- |
| ***Histology finding*** | | | ***n (%)*** | | |
|  | **Malignancy (Carcinoma)** | | **73 (56.6)** | | |
|  |  | Adenocarcinoma lung | | 44 | |
|  |  | Squamous cell carcinoma | | 4 | |
|  |  | Small cell carcinoma | | 8 | |
|  |  | Metastatic adenocarcinoma breast | | 3 | |
|  |  | Metastatic adenocarcinoma | | 3 | |
|  |  | Metastatic clear cell renal carcinoma | | 2 | |
|  |  | Non-small cell carcinoma NOS | | 2 | |
|  |  | Adenosquamous carcinoma | | 2 | |
|  |  | Large cell neuroendocrine carcinoma | | 1 | |
|  |  | Pleomorphic sarcomatoid carcinoma | | 1 | |
|  |  | Metastatic hepatocellular carcinoma | | 1 | |
|  |  | Metastatic nasopharyngeal carcinoma | | 1 | |
|  |  | Metastatic adenocarcinoma endometrial | | 1 | |
|  | **Malignancy (Rare)** | | **3 (2.3)** | | |
|  |  | Classic Hodgkin lymphoma | | | 2 |
|  |  | Germ cell tumour (choriocarcinoma) | | | 1 |
|  | **Benign Tumour** | | **2 (1.6)** | | |
|  |  | Oesophageal leiomyoma | | | 1 |
|  |  | Sclerosing pneumocytoma | | | 1 |
|  | **Non Caseating Granuloma** | | **11 (8.5)** | | |
|  |  | Sarcoidosis | | | 6 |
|  |  | Tuberculosis (therapeutic response to anti-tuberculous therapy) | | | 2 |
|  |  | Sarcoid-like reaction (underlying breast cancer) | | | 1 |
|  |  | Eosinophilic lung disease | | | 1 |
|  |  | Infective (Salmonella sepsis) – on surveillance | | | 1 |
|  | **Caseating Granuloma** | | **6 (4.7)** | | |
|  |  | Tuberculosis | | | 6 |
|  | **Lymphoid Tissue or Inflammatory** | | **19 (14.7)** | | |
|  |  | Inflammatory (stable or resolved on surveillance imaging) | | | 15 |
|  |  | Reactive (proven by VATS) | | | 2 |
|  |  | Silicosis | | | 1 |
|  |  | IgG4 related disease | | | 1 |

| **Supplementary Table 4 Histology of inconclusive EBUS-TBMC cohort (N=15)** | | | |
| --- | --- | --- | --- |
| ***Histology*** | | ***n (%)*** | |
| **Inadequate sampling** | | **13 (86.7)** | |
|  | Lymphoid tissue | | 5 |
|  | Necrotic tissue | | 4 |
|  | Crushed lymphocytes with carbon deposit | | 2 |
|  | Fibrotic tissue | | 1 |
|  | Lymphoid tissue with sinus histiocytosis | | 1 |
| **Sampling error** | | **2 (13.3)** | |
|  | Lung tissue with lymphocytic infiltration | | 1 |
|  | Thymic tissue | | 1 |

| **Supplementary Table 5 Final outcome of cohort with inconclusive EBUS-TBMC (N=15)** | | | |
| --- | --- | --- | --- |
| ***Final outcome*** | | ***n (%)*** | |
| **Defaulted or Refused Re-biopsy** | | **5 (33.3)** | |
| **Malignancy (Carcinoma)** | | **3 (20.0)** | |
|  | Adenocarcinoma lung (*via CT guided biopsy*) | | 2 |
|  | Pleomorphic carcinoma with squamous differentiation (*via repeat EBUS/TBNA*) | | 1 |
| **Benign Tumour** | | **2 (13.3)** | |
|  | Thyroid nodular hyperplasia (via thyroidectomy) | | 1 |
|  | Sclerosing pneumocytoma (via VATS) | | 1 |
| **Sarcoidosis** | | **2 (13.3)** | |
|  | Potential sarcoid-like reaction (underlying adenocarcinoma lung) | | 1 |
|  | Clinically high provisional confidence diagnosis (high serum ACE level) | | 1 |
| **Tuberculosis** | | **1 (6.7)** | |
|  | Repeated bronchoalveolar lavage positive Xpert MTB/RIF | | 1 |
| **Malignancy (Rare)** | | **1 (6.7)** | |
|  | Germ cell tumour (via diagnostic VATS) | | 1 |
| **Rare Lung Disease** | | **1 (6.7)** | |
|  | Castleman disease (via diagnostic VATS) | | **1** |

| **Supplementary Table 6 Correlation of TBMC specimen size to overall diagnostic yield** | | | | |
| --- | --- | --- | --- | --- |
|  | | | | *p-value* |
| Median total aggregate diameter (IQR), *mm* | | *Conclusive* | 7.0 (5.0-10.0) | 0.004 |
|  |  | *Inconclusive* | 5.0 (4.0-7.0) |  |
| Correlation of specimen size to conclusive procedure, *r* | | | + 0.240 | 0.006 |
| Association with diagnostic yield in different specimen size thresholds, *n (%)* |  | *<4 mm* | 2/4 (50.0) | 0.015 |
|  |  | *≥4 mm* | 112/125 (89.6) |  |
|  |  | *<5 mm* | 5/9 (55.6) | 0.001 |
|  |  | *≥5 mm* | 109/120 (90.8) |  |
|  |  | *<6 mm* | 33/43 (76.7) | 0.004 |
|  |  | *≥6 mm* | 81/86 (94.2) |  |
|  |  | *<7 mm* | 50/61 (82.0) | 0.032 |
|  |  | *≥7 mm* | 64/68 (94.1) |  |
|  |  | *<8 mm* | 69/81 (85.2) | 0.142 |
|  |  | *≥8 mm* | 45/48 (93.8) |  |
|  |  | *<10 mm* | 83/97 (85.6) | 0.084 |
|  |  | *≥10 mm* | 31/32 (96.9) |  |
|  |  | *<12 mm* | 108/123 (87.8) | 0.363 |
|  |  | *≥12 mm* | 6/6 (100.0) |  |

| **Supplementary Table 7 Correlation of TBMC cryo-pass to specimen size and diagnostic yield** | | | | |
| --- | --- | --- | --- | --- |
|  | | | | *p-value* |
| Median cryo-pass (IQR), *passes* | | *Conclusive* | 4 (3-4) | 0.028 |
|  |  | *Inconclusive* | 3 (2-4) |  |
| Correlation of cryo-pass to specimen size, *r* | | | + 0.254 | 0.004 |
| Correlation of cryo-pass to conclusive procedure, *r* | | | + 0.196 | 0.026 |
| Association with diagnostic yield in different cryo-pass, *n (%)* |  | *<2 passes* | 1/3 (33.3) | 0.003 |
|  |  | *≥ 2 passes* | 113/126 (89.7) |  |
|  |  | *<3 passes* | 12/16 (75.0) | 0.075 |
|  |  | *≥ 3 passes* | 102/113 (90.3) |  |
|  |  | *<4 passes* | 52/63 (82.5) | 0.043 |
|  |  | *≥ 4 passes* | 62/66 (93.9) |  |
|  |  | *<5 passes* | 96/110 (87.3) | 0.349 |
|  |  | *≥ 5 passes* | 18/19 (94.7) |  |
|  |  | *<6 passes* | 110/124 (88.7) | 0.551 |
|  |  | *≥ 6 passes* | 4/5 (80.0) |  |

| **Supplementary Table 8 Correlation of TBMC freezing time to specimen size and diagnostic yield** | | | | |
| --- | --- | --- | --- | --- |
|  | | | | *p-value* |
| Median freeze time per pass (IQR), *seconds* | | *Conclusive* | 7.0 (5.5-8.8)) | 0.314 |
|  |  | *Inconclusive* | 8.0 (6.0-10.0) |  |
| Correlation of freeze time per pass to specimen size, *r* | | | - 0.040 | 0.656 |
| Correlation of freeze time per pass to conclusive, *r* | | | - 0.052 | 0.558 |
| Association with diagnostic yield in different freezing time, *n (%)* |  | < 4 s | 8/8 (100) | 0.289 |
|  |  | ≥ 4 s | 106/121 (87.6) |  |
|  |  | < 7 s | 56/60 (93.3) | 0.101 |
|  |  | ≥ 7 s | 58/69 (84.1) |  |
|  |  | < 10 s | 91/102 (89.2) | 0.561 |
|  |  | ≥ 10 s | 23/27 (85.2) |  |
|  |  | < 13 s | 106/120 (88.3) | 0.960 |
|  |  | ≥ 13 s | 8/9 (88.9) |  |

| **Supplementary Table 9 Diagnostic yield of EBUS-TBNA vs. TBMC in common malignancy** | | |
| --- | --- | --- |
| ***Procedure*** | ***Diagnostic yield, n/N (%)*** | ***p-value*** |
| EBUS-TBNA | 33/55 (60.0) | 0.078 |
| EBUS-TBMC | 53/55 (96.4) |  |

| **Supplementary Table 10 Diagnostic yield of EBUS-TBNA vs. TBMC in common malignancy** | | | |
| --- | --- | --- | --- |
|  | TBMC Conclusive | TBMC Inconclusive | *Total* |
| TBNA Conclusive | 33 | 0 | *33* |
| TBNA Inconclusive | 20 | 2 | *22* |
| *Total* | *53* | *2* | *55* |

| **Supplementary Table 11 Diagnostic yield of EBUS-TBNA vs. TBMC in uncommon tumours and benign disorders** | | |
| --- | --- | --- |
| ***Procedure*** | ***Diagnostic yield, n/N (%)*** | ***p-value*** |
| EBUS-TBNA | 20/41 (48.8) | 0.005 |
| EBUS-TBMC | 34/41 (82.9) |  |

| **Supplementary Table 12 Diagnostic yield of EBUS-TBNA vs. TBMC in uncommon tumours and benign disorders** | | | |
| --- | --- | --- | --- |
|  | TBMC Conclusive | TBMC Inconclusive | *Total* |
| TBNA Conclusive | 20 | 0 | *20* |
| TBNA Inconclusive | 14 | 7 | *21* |
| *Total* | *34* | *7* | *41* |
